# The quest to share data

**DOI:** 10.3389/fninf.2025.1570568

**Published:** 2025-03-19

**Authors:** Arthur W. Toga, Sidney Taiko Sheehan, Tyler Ard

**Affiliations:** Laboratory of Neuro Imaging, Stevens Neuroimaging and Informatics Institute, Keck School of Medicine of USC, University of Southern California, Los Angeles, Los Angeles, CA, United States

**Keywords:** data sharing, re-usability, FAIR principles, repositories, infrastructure, guidelines, privacy

## Abstract

Data sharing in scientific research is widely acknowledged as crucial for accelerating progress and innovation. Mandates from funders, such as the NIH’s updated Data Sharing Policy, have been beneficial in promoting data sharing. However, the effectiveness of such mandates relies heavily on the motivation of data providers. Despite policy-imposed requirements, many researchers may only comply minimally, resulting in data that is inadequately reusable. Here, we discuss the multifaceted challenges of incentivizing data sharing and the complex interplay of factors involved. Our paper delves into the motivations of various stakeholders, including funders, investigators, and data users, highlighting the differences in perspectives and concerns. We discuss the role of guidelines, such as the FAIR principles, in promoting good data management practices but acknowledge the practical and ethical challenges in implementation. We also examine the impact of infrastructure on data sharing effectiveness, emphasizing the need for systems that support efficient data discovery, access, and analysis. We address disparities in resources and expertise among researchers and concerns related to data misuse and misinterpretation. Here, we advocate for a holistic approach to incentivizing data sharing beyond mere compliance with mandates. It calls for the development of reward systems, financial incentives, and supportive infrastructure to encourage researchers to share data enthusiastically and effectively. By addressing these challenges collaboratively, the scientific community can realize the full potential of data sharing to advance knowledge and innovation.

## 1 Introduction

Data sharing holds the promise to accelerate progress and innovation in scientific discovery and is increasingly required through funder-initiated mandates. However, without an environment where providers are sufficiently incentivized to share data enthusiastically through systems that support practical reusability, the full potential of data sharing to transform modern science will not be realized. Many scientific communities have embraced the idea that open science and freely sharing data can further our quest to derive information and knowledge. Furthermore, governmental funding agencies and private foundations generally embrace the concept of open science and increasingly require data sharing when research award are granted. Examples of this can be seen in Horizon Europe’s open science requirements (European Commission, 2021), UK Research Innovation (UKRI) open access policy (UK Research and Innovation, 2020), the Wellcome Trust’s Data, software, and materials management and sharing policy (Wellcome Trust, n.d.), and newly mandated National Institutes of Health (NIH) Data Management and Sharing (DMS) requirements (National Institutes of Health, n.d.).

Such policy-imposed mandates take an important step in requiring investigators to comply regardless of their desire to contribute. Still, without fully cooperative and enthusiastic investigators, these efforts may fail to have the desired impact. Ultimately, the minimum effort required for compliance often results in minimally reusable data, as no oversight can perfectly define, monitor, and verify all the details necessary for successfully sharing and reusing data across the myriad of investigators, projects, and databases. The impact of data sharing is also strongly modulated by both the physical infrastructure and software it is shared through, which are typically selected by contributors and rarely standardized. As such, we must consider not just what rules should be put in place but also how the overall system can be designed to create compelling incentives to significantly motivate providers to share data in an effective, reusable manner.

There are many complex issues to address if we are to establish an environment that maximizes the impact of data sharing. We do not have clear answers to these issues, and this paper is in no way intended to be prescriptive, although inevitably, personal biases may be evident. Rather, this is an attempt to raise issues that will not be sufficiently addressed via mandates that have been heard and witnessed by colleagues with whom we have worked as part of our academic informatics center and programs over the years.

## 2 Data, guidelines, and stakeholders

When considering data sharing broadly, we must acknowledge that the goals of data sharing differ between funders, data providers, users, regulators, participants, and patients. There are differences across cultures and countries. There are differences between commercial concerns and academics. There are even differences between scientific disciplines. Each constituent has a different perspective, and we must acknowledge this and account for their respective motivations and concerns. Here, while we do discuss some individual examples, we mainly focus on ubiquitous elements and factors across broad sections of the scientific community.

### 2.1 The funders

While there are many commendable efforts to establish and improve the sharing of data for the furtherment of scientific progress, funder-initiated mandates are particularly notable for their potential to expand the breadth of data sharing dramatically. Funder goals for data sharing likely include maximizing the value, significance, and contribution of the science (and data) produced by funded projects. Reusing data is a more efficient use of time and resources and can reduce research cost (Meystre et al., 2017). Funders may want to ensure the funding source is well and clearly represented in all publications, including those derived from the data reuse. It should also be noted that funders may want to make certain that the re-distribution of data is limited or prohibited to manage data integrity and adherence to data use stipulations.

A few data sharing workshops have called for alterations to systems in academic research to facilitate cohort data sharing. Creating and managing cohorts requires effort and resources from numerous specialists collaborating effectively over time, which is not often recognized by academic reward systems (Devriendt et al., 2023). Over the years, countless discussions, workshops, and “white papers” on modifying academic review in university promotion processes to include cohort data sharing have occurred, with little consequence.

### 2.2 The guidelines

As science has evolved into a more data-driven economy, it has become clear that there are significant complexities to sharing data beyond those related to technology and infrastructure. Existing guidelines, such as those enumerated by the FAIR (Findable, Accessible, Interoperable, and Reusable) principles, provide beneficial goals for good data management and stewardship (Wilkinson et al., 2016). These principles effectively facilitate the efforts of those dedicated to data sharing, and commendable communities are utilizing them to great effect. However, without certain sociological, practical, and ethical considerations, mere guidelines may fail to achieve the intended data sharing goals fully. Notably, for many researchers, creating and reorganizing data according to FAIR principles represents a considerable use of time and resources that they would rather allocate to other efforts. It is this broad audience that new funder-initiated mandates impact and that proper motivations could prove particularly effective. Our goal here is not to disparage researchers who are not data-sharing enthusiasts but rather recognize and frankly discuss factors that discourage any researcher from freely and fully committing to open data sharing.

### 2.3 The providers

The investigator who collects data based upon carefully designed protocols, balanced and appropriate subject recruitment, consistent methodology, and comprehensive documentation to answer hypotheses described in (often multiple applications before success) a funded grant and who may or may not yet have analyzed and written the paper describing the findings, rightfully has some concerns regarding the when, what, and how those data are shared. It is also understandable that investigators may have a sense of ownership and desire for (some) proprietary control. Through our work, we have recognized that many investigators do not want to merely hand over the data they spent considerable resources and time collecting. Others have found similar attitudes as measured by surveys on open data (Goodey et al., 2022). Furthermore, when data are collected from human subjects, the concept of “ownership” is more complex (see section “3.4 Compliance, regulation, and legal considerations”), especially when information that could identify the subject is included or linked. There are legal and compliance issues that can restrict data sharing or require additional precautions that can also diminish a provider’s enthusiasm for sharing the data.

### 2.4 The data

When broadly considering scientific data, we must acknowledge that not all data is of equal value, and the mere presence of accessible data does not speak to its worthiness (Schmidt et al., 2021). There is currently little differentiation between data that has been diligently organized, documented, and arranged for harmonization compared to data that has received little to no reuse optimization. Simply stated, disorderly, inconsistent, and inadequately described data can comply with data sharing requirements.

We must also consider what should be provided in the data sharing process. Quality control metrics, complete provenance, and metadata can enhance both the primary utilization of the data and subsequent and unanticipated reuse. Adopting standards, when appropriate, and using carefully curated ontologies, detailed dictionaries, and accepted common data elements are essential to achieve the broadest possible data sharing. Notably, these and other factors that contribute to creating data that is effective for reuse have been known for many years and are encouraged by funders. However, while the content and structure of reusable data can appear simple at a broad level, the specific details necessary for effectively reusing an individual dataset can be highly nuanced, often to the extent that it is only known by those intimately involved with the data.

Compounding this issue, we do not have standardized systems to evaluate data sets’ potential benefits. The so-called “vote with your feet” approach provides an obvious solution, where datasets are scored by the number of times they are downloaded and/or utilized in further publications. However, it should be noted that while sharing data through a repository that provides the ability to measure attribution, citation, and reuse of data is often encouraged by funders, it is generally not required even when data sharing itself is. This raises the question of how effectively we can measure the impact of our efforts. This issue is further complicated by numerous repositories that lack standardization of both tracking methods and the means of disclosing those methods.

## 3 Considerations for data sharing

When considering the difficulties evaluating shared data, it becomes apparent that a motivated data provider willing to dedicate substantial effort to the cause is instrumental in facilitating the ultimate effectiveness of shared data. However, establishing an environment that broadly incentivizes data sharing to motivate providers sufficiently represents a considerable challenge. But, regardless of the level of difficulty, this is a challenge we must rise to meet as sole reliance on compliance will drive contributors to be more concerned with the letter of the law than the spirit of it, undoubtedly resulting in unforeseen consequences and suboptimal outcomes. While true motivation would certainly be dramatically more effective than forced compliance universally, this difference should be expected to be even more pronounced across the numerous scientific studies and disciplines that produce vast and complex forms of data, where a multitude of factors, both small and large, considerably impact effective reuse. FAIR principles and additional discipline-specific standards can provide requirements to improve the reusability of shared data. However, it seems unlikely that guidelines can ever comprehensively account for the myriad of nuanced factors that must be meticulously addressed to support the effective reuse of various forms of scientific data, which can vary on a study-by-study basis.

Effective data sharing can be complex to the point that even when providers are motivated enthusiasts, it still proves difficult to properly document and arrange in a manner supportive of data harmonization, reanalysis, and meta-analysis. If providers are solely compelled by mandate, many shared datasets will likely not receive the attention and rigor required to make them effectively reusable, ultimately resulting in many shared datasets but few broader impacts.

Lastly, sole reliance on compliance implies the existence of enforcement methods that verify data is provided in a fully reusable fashion. However, establishing such effective enforcement is unfeasible due to the factors discussed above, as well as numerous additional considerations. Who would provide the judgment of the reusability of the data? It is unlikely that the largely unpaid article reviewers would tolerate the additional duties of inspecting and verifying that all shared data is properly arranged, meta-tagged, and effectively reusable. Alternatively, placing the burden of data verification on the repositories that store it would prove problematic for numerous reasons, including funding, qualification, and bias.

Furthermore, if repositories were to police data, measures would need to be implemented to prevent providers from simply sharing their data through the least rigorous repository or providing data through an unsupervised generic hosting service. Additional layers of regulation to force the use of specific approved repositories would require vastly more oversight and introduce a rigidity that would prove difficult to adjust to new and emerging forms of data rapidly. Ultimately, attempting to establish effective enforcement for data sharing may very well create more problems than it would solve.

### 3.1 Data sharing incentives

Investigators may be more enthusiastic about sharing data if authorship on the paper that results from the data sharing is promised, with some advocates calling for publishers to require authors who use data generated by other researchers to be acknowledged as “data authors” and others calling for independent dataset digital object identifiers (DOIs) to be more reliably used as additional citations (Hughes et al., 2023). Investigators may be even more eager to share data after their own papers have been published. Investigators, laboratory members, centers, institutes, universities, and others often want to be acknowledged. There are many models to achieve this. Data Use Agreements often stipulate the type of acknowledgment, including authorship or listing as a collaborator in the author list, for example.

Investigators may be more motivated by positive performance, such as if the data shared results in confirmation of their findings as opposed to contradicting them. Confirmation of their findings could lead to new opportunities, while contradictory findings may perpetuate a fear of loss of data autonomy. This fear is understandable, as in many cases, independent analysis of data often produces different results (Oza, 2023). The possible negative impacts that researchers can experience from data sharing are problematic, as they cannot be addressed in a manner that compromises scientific integrity. Acknowledging this situation is often unacceptable, as it reflects researcher motives that can be at odds with scientific rigor. However, we feel this factor should be discussed as it can significantly impact data sharing, particularly as the risk of contrary findings outweighs the benefits of additional citations and authorship. Surveys and analysis of factors influencing data sharing frequently reveal that investigators claim to be more willing to share if the benefits in future grant getting, reputation, appreciation, and even academic recognition are provided (Zuiderwijk et al., 2020).

As data sharing requires an ever-increasing abundance of time and resources, incentives beyond citations and visibility may be required to prioritize data sharing. What reward systems can be put into place to motivate data contributors effectively? Science is conducted primarily within a competitive system to receive funding; thus, using financial incentives to promote data sharing and reuse in mutually beneficial systems may prove useful. For example, we could utilize a similar approach to the funders experimenting with prizes to reward investigators who generate a novel finding, tool, or application from existing data (MacFarlane, 2022), but targeted instead to those who provide the data. Small Wellcome trust data re-use prizes (MacFarlane, 2022) begin to explore this territory, but allocating more significant funding for additional data collection to those who offer broadly utilized datasets could be a potent tool to fuel effective data sharing.

While an increase in investigator motivation to share data when positive reinforcements are in place may seem like an obvious conclusion, it remains a complicated situation.

### 3.2 Analytic and support disparities

Investigators might be wary of sharing data if disparities in resources are apparent. What if data recipients have an overwhelming capacity for rapid and sophisticated analysis compared with the data provider? Considering that around half of investigators frequently use data generated by other researchers, we know this dynamic is already taking place (Tedersoo et al., 2021). This presents a more complicated question we have yet to see addressed on a more significant scale. Lack of reciprocity illustrates how data producers may feel exploited, while data users may advance significantly in their research and careers, which may further exacerbate existing disparities in resources (van Panhuis et al., 2014).

Additionally, we must consider that in many cases, sharing the data itself can be a significant burden where infrastructure and data sharing expertise are underdeveloped. Indeed, surveys of investigators show factors such as “lack of time to deposit data” and “costs of sharing data” are notable challenges, particularly with under-resourced researchers and institutions (Stuart et al., 2018).

### 3.3 Use, misuse, and misinterpretation

No one can predict all the ways a dataset can be potentially worthwhile in future investigations. As such, there is often significant ambiguity surrounding the issue of how data is reused, which can be problematic when considering the potential for data to be misused and/or misinterpreted. Data use agreements may attempt to regulate how a dataset can be reused strictly but at the cost of limiting the usefulness of that dataset to narrowly defined definitions. This issue is further complicated when considering that there is no guarantee that data use agreements will be adhered to or avenues to pursue correction or recompense if they are broken. A systematic approach to addressing these concerns could encourage more data sharing while keeping researchers accountable for their work. Trust between data providers and users could facilitate complete and effective data sharing, but how can we systemically create trust? Trust relationships are often made through longstanding collaboration and mutually beneficial exchanges. Legal arrangements may be necessary to develop a foundation of trust when previous positive relationships among data providers and users are absent.

Further, data providers may take issue with the intended secondary use of their data. Ethical disagreements could arise between groups regarding the proportional risks and benefits of the secondary use of data (Romain, 2015). Defining how these situations are to be mediated would be an important addition to legal arrangements between data providers and users.

### 3.4 Compliance, regulation, and legal considerations

The current legal and regulatory framework regarding open data sharing directly and severely limits the effective sharing of data. The majority of the components in these frameworks are understandably focused on protecting the data and the participants, if human. However, there is a striking lack of such considerations for the investigator, which is particularly troubling when combined with the lack of assurance that any Data Use Agreement is adhered to. We should consider regulations to absolve data producers in situations such as data misuse resulting in harmful clinical recommendations. Additionally, mechanisms that assure the data use agreement will be adhered to would no doubt be welcomed to prevent misuse and verify that proper credit is given to the provider, among numerous other reasons.

Furthermore, the legal landscape, as well as regulatory and privacy concerns, are complex and constantly evolving. Those fearing liability may interpret regulations in a restrictive way and be understandably less likely to share data, particularly if they lack institutional legal guidance and support (Eke et al., 2022). We already see how legal uncertainty from data providers creates ambiguity, ultimately impacting individual researchers (Geneviève et al., 2021). Data sharing requirements are driven by policy and, in many instances, motivated by the need to limit liability, both legal and financial, as well as maintain positive public perception and reputation.

Additional concerns of privacy and data ownership are particularly acute in studies involving human subjects, where participants also have changing expectations for how, where, and when data about them can be used (White et al., 2022). These considerations, along with complex issues of data-ownership of biomedical human subject data, vary significantly across institutions, funding agencies, and countries in a manner that is further nuanced by factors such as subject age and if the data is fully anonymized or de-identified/pseudonymized. Adding to this intricate web of considerations is the fact that these diverse regulations change considerably over time.

How can we comply with rules that change? Once data has been shared and rules become more restrictive, there are no means to recall data that no longer complies effectively. It can be incredibly complex for investigators to adhere to their respective legal, privacy, and regulatory requirements while sharing some form of data. Related issues are addressed in the sharing of intellectual property through general-purpose license agreements such as creative commons. Implementing a similarly broad strategy for research data could prove beneficial.

### 3.5 When to share data?

The fundamental question of when data should be shared has many unaddressed considerations that severely affect the impact of shared data as well as the level of burden it places on the contributor. Suppose an investigator is solely motivated by required compliance. In that case, they may choose to share data prior to (or without) performing any quality control, augmentation in the form of provenance, dictionary definitions, or processing critical to its effective use. Even in cases where a researcher truly desires to share data in a reusable way, it is often unclear if it is best shared raw, processed, or both at the cost of significant additional hosting resources.

Furthermore, quality should also be considered when evaluating when to share data. Take, for example, the recent data from the COVID-19 pandemic. While rapid data sharing in COVID-19 research was essential to facilitate prompt assessment of the safety and efficacy of vaccines, some of the first published articles on COVID-19 had to be withdrawn because of quality issues. It should also be noted that even with accelerated data sharing in COVID-19 research, the sharing of reusable data from open access articles was still low—of papers indexed in the PMC, deposit of supplementary material and data in repositories was only 13.6%, and reusable data was merely 1.2% (Lucas-Dominguez et al., 2021). While the speed of COVID-19 research undoubtedly contributed to the low amount of reusable data, it is by no means the only factor. Instead, rapid COVID-19 research was already occurring in an environment where many researchers were not effectively motivated to dedicate the time and effort necessary to share their research data.

### 3.6 Infrastructure

While this article focuses on the motivation of researchers and how that motivation will have a profound effect on the impact of shared data, there is an additional consideration beyond motivation that should be considered. Specifically, once motivation is established, a researcher must select or develop the infrastructure through which to share their data. This infrastructure also strongly affects the impact of shared data, to the point that it is often a primary topic in policy discussion. However, many unanswered questions remain beyond the scope of individual researchers regarding how such infrastructure can be designed to optimize the overall impact of shared data.

What types of database systems, schemas, and security measures are best suited? What types of management systems can be created? Is the specific scientific community adequately served, and can we tailor each implementation to the needs of that discipline? Should data infrastructures be centralized, linked, or federated? No single solution can possibly fit all needs. Do we have the resources to develop specialized systems? And how could they be interoperable? We see substantial differences in preferences from stakeholders regarding control, consent, incentivization, useability, and trust in technology (Hermansen et al., 2022).

The cloud is often touted as the solution to storing and computing shared data, but have the costs been truly assessed? Depending on the type of data, how often it changes, whether data for a project is still being collected, whether users want or expect to be able to download the data, how large and how many files exist and of what type, and the computational load of analysis are all cost-determining factors. Currently, it is unclear who pays, and when, and what is financially equitable. Further, legal barriers complicate the implementation of cloud-based solutions, particularly if cloud servers are hosted in jurisdictions with regulatory frameworks that differ from those governing the data’s origin. For example, storing and processing GDPR compliant data in a country with less robust privacy protection can introduce unforeseen risk and legal ramifications.

While cloud-based storage provides benefits such as relative ease of access, scalability, etc., implementation must address identity management issues, access control, contractual obligations, and longevity. How long is the data to be made available? Forever? Cloud-based solutions are for-profit companies. Is that a problem, and if not now, can it become one? Is domain expertise needed, and can these companies provide it? How might the motivations of cloud service providers differ from data providers, funding agencies, and data users? For these reasons, many investigators feel the need to retain possession of the data and handle sharing personally or work with an outside trusted investigator for assistance. Often, the implementation of the sharing infrastructure, such as storage, search, access control, distribution, logging, support, and other aspects, can only be achieved by informatics experts in the same or similar scientific discipline. With large data sets, co-localizing compute capabilities with data avoids transmission across limited bandwidth networks. Does this restrict large datasets to cloud-based solutions? There are countless data centers at universities that are not fully utilized. Can these contribute?

The challenge is not just storing the data but also finding it. Search is often complex, especially in disciplines and studies that utilize numerous variables. Within a data storage system, data are only usable if a researcher can search and retrieve them, make sense of them, and analyze them within a single study or combine them across multiple studies. Thus, data must be in a computable form, amenable to standardized and automated methods of search, analysis, and visualization. These capabilities are supported by metadata, which allows researchers to discover datasets and evaluate their usefulness in tandem (Tsueng et al., 2023). Interoperability of shared data should enable data aggregation from multiple studies and meta-analyses across them, supported by an underlying cross-study searching capability. There are many US-based repositories emerging in response to the NIH Data Sharing Policy which went into effect last year, in addition to several prominent European repositories. However, as noted by survey (Shearer et al., 2023), existing repositories already have significant challenges related to meta-data cohesiveness, infrastructure, visibility, and interoperability. Should we consider how navigable the fractured and highly diverse data sharing landscape will be for researchers and if it will ultimately impact the effective reuse of data?

Expertise in informatics, machine learning, and software development is often required to develop data sharing infrastructure in a manner that can be efficiently utilized. It should be noted that building an infrastructure, regardless of where it is located, is only the beginning. Systems invariably require constant updating, modification, and adaptation to new requirements. Many researchers are not explicitly trained in these areas, creating a knowledge gap. This could impact not only the proper collection of data but also data sharing and appropriate use of data shared. How do we account for disparities in training? Successful infrastructure should also include those who wield the tools. Support for data experts, or even training for non-experts, should be factored in (Hughes et al., 2023).

Lastly, we should consider how infrastructure makes data available, and to whom. Scientific data typically requires specialized software and technical capabilities to view or interact with, naturally limiting accessibility to analytic researchers already familiar with that form of data. This exclusivity is evident in measurements that show digital supplementary material access rates below 0.04% of principal articles with supplements (Flanagin et al., 2018), indicating they are simply not utilized by the general readership. Previous approaches such as Wiley’s Anywhere Article (Wiley, 2014) and Elsevier’s Article of the future (Elsevier, 2012) as well as new technologies such as Schol-AR (Ard et al., 2022) aim to integrate scientific data viewing capabilities directly into articles themselves, providing accessible data to all readers. Would it be beneficial to bridge our emerging data sharing infrastructures with these types of technologies to provide ready access to all readers, even if they will not further analyze the data?

## 4 Discussion

The concept of data sharing is positive at most every level. The dissemination and communication of the knowledge gained from scientific studies are vital, and the value of combined data provides power to many investigations. Open access data can also bring together researchers from many complementary and adjacent disciplines who might not otherwise have the opportunity to collaborate and further each other’s work.

Mandates are an important step in expanding the breadth and scope of data sharing. However, if investigators share data solely because they are forced to, the desired result of data sharing may remain unrealized. An unmotivated investigator can comply with data sharing mandates while using an ineffective infrastructure, with data not properly prepared for effective reuse or without one of the many elements necessary for interpretation. These and many other barriers that hinder useful data resharing can occur even when an investigator is not attempting to restrict data reuse but instead simply desires to check the compliance box while not putting forth the substantial effort required to share data effectively.

To broadly motivate contributors to share data earnestly and laboriously, we must create an incentivized environment that synergizes with mandates. This will be difficult. There are many complex factors underlying both the incentives and deterrents that impact contributor motives. It is unlikely we will develop a perfect solution at first, or possibly ever. But certainly, researchers across the scientific spectrum can come together to help find innovative, effective, and most importantly, welcomed solutions for significantly enhancing the motivation for data sharing. Mandates alone do not facilitate discovery, and without addressing the challenges noted above, it will be difficult to improve the data sharing process to allow investigators to unite globally and address scientific questions of great complexity.

## Data Availability

The original contributions presented in this study are included in this article/supplementary material, further inquiries can be directed to the corresponding author.
